# FvNST1b NAC Protein Induces Secondary Cell Wall Formation in Strawberry

**DOI:** 10.3390/ijms232113212

**Published:** 2022-10-30

**Authors:** Xiaofei Dang, Bei Zhang, Chen Li, Shingo Nagawa

**Affiliations:** 1 College of Horticulture, Fujian Agriculture and Forestry University, Fuzhou 350002, China; 2 Fujian Agriculture and Forestry University–University of California, Riverside, Joint Center for Horticultural Biology and Metabolomics, Haixia Institute of Science and Technology, Fujian Agriculture and Forestry University, Fuzhou 350002, China

**Keywords:** FvNST1b, secondary wall thickening, strawberry, *Arabidopsis*

## Abstract

Secondary cell wall thickening plays a crucial role in plant growth and development. Diploid woodland strawberry (*Fragaria vesca*) is an excellent model for studying fruit development, but its molecular control of secondary wall thickening is largely unknown. Previous studies have shown that *Arabidopsis* NAC secondary wall thickening promoting factor1 (AtNST1) and related proteins are master regulators of xylem fiber cell differentiation in multiple plant species. In this study, a NST1-like gene, *FvNST1b*, was isolated and characterized from strawberry. Sequence alignment and phylogenetic analysis showed that the FvNST1b protein contains a highly conserved NAC domain, and it belongs to the same family as AtNST1. Overexpression of *FvNST1b* in wild-type *Arabidopsis* caused extreme dwarfism, induced ectopic thickening of secondary walls in various tissues, and upregulated the expression of genes related to secondary cell wall synthesis. In addition, transient overexpression of *FvNST1b* in wild-type *Fragaria vesca* fruit produced cells resembling tracheary elements. These results suggest that FvNST1b positively regulates secondary cell wall formation as orthologous genes from other species.

## 1. Introduction

The secondary cell wall (SCW) is typically composed of lignin, cellulose, and hemicelluloses (xylan and glucomannan and galactoglucomannans). The SCW is formed inside the primary cell wall after the cell is fully expanded. SCW structures have large impacts on the characteristics of plant cells and organ development and play important roles in the dehiscence of anthers and silique pods, enhance mechanical support of organs, facilitate water transport, and provide a barrier against invasive pathogens [1,2,3,4]. The SCW is characteristically formed in xylem vessels and fibers and is crucial in the development of secondary xylem. The deposition of secondary walls reinforces stability of these cells, allowing them to provide structural support and protection [5]. The secondary walls of anther endothecium have striated patterns similar to those in tracheary elements. These secondary wall thickenings are necessary for anther dehiscence, and they generate the tensile force necessary for the rupture of the stomium [6]. Lignification in the endodermal layer of the valve margin of silique pods is necessary for their dehiscence, generating tension via desiccation and leading to pod shattering [7,8,9].Thus, SCW provides crucial biological roles in various organs, and unveiling mechanisms behind the regulation of SCW formation has been an important topic in the plant developmental research.

Extensive studies have been performed in multiple species from angiosperms such as *Arabidopsis* and *Zinnia elegance* to resolve the transcriptional network controlling xylem vessel differentiation and SCW formation. Several proteins in the plant-specific NAC (NAM, ATAF1/2 and CUC2) transcription factor (TF) family have been found to play a central role in this process [4,10,11]. NAC TFs have been found to play important roles in plant development and environmental responses [12,13,14,15,16]. Members of this family have been reported to be involved in the ripening and softening of fleshy fruits such as citrus [17], tomato [18,19], peach [20], kiwifruit [21], and apple [22]. These TFs share an NAC domain consisting of 130–150 amino acids at their N-terminus that allows for their interaction with DNA. The NAC domain is further divided into five subdomains, A–E, from N-terminus to C-terminus, respectively [23]. NAC TFs also contain a transcriptional regulatory (TR) domain at the C-terminal region, and some NAC TFs have a transmembrane domain within the TR domain [24].

NST1, originally identified in *Arabidopsis*, is a member of the NAC domain transcription factor family with a highly conserved N-terminal NAC domain [2]. NST1 and NST2 act redundantly to regulate the SCW thickenings in anther walls [2], and NST1 and NST3 (also called SND1/ANAC012) redundantly regulate the SCW thickenings in interfascicular fibers of inflorescence stems, secondary xylem of hypocotyls, and silique cells [25]. During anther development, MYB26 plays a regulatory role in determining cell development, or competence for secondary thickening, and acts upstream of the lignin biosynthesis pathway presumably via NST1 and NST2 [2]. NST1 promoter activity was detected in various tissues in which lignified secondary walls develop [2]. Recent studies have shown that NSTs and orthologs positively and coordinately regulate SCW formation in several plant species [26,27,28]. Thus, NST1 is considered a master regulator for SCW formation [2,14]. Among NAC proteins, some closely related members including VND, NST, SMB, and BRN of *Arabidopsis* have been termed the VNS (VND, NST/SND, SMB related protein) family [29,30]. Vascular-related NAC-DOMAIN1-7 (VND1-7) proteins regulate xylem differentiation in *A. thaliana* [11]. VND6 and VND7 are determinants of tracheary element differentiation which is also associated with SCW deposition [11,31,32,33], whereas VND1-VND5 might induce tracheary element differentiation indirectly, for example, by inducing the expression of VND6 and VND7 [31]. VND-related *Arabidopsis* proteins, namely SOMBRERO (SMB), BEARSKIN1 (BRN1), and BRN2, induce ectopic SCW deposition in many cell types when overexpressed, such as root, hypocotyl, shoot meristem, and cotyledon [34,35]. Thus, VNSs have a common role to promote SCW deposition.

Functional analysis of VNS homologs from various plant species, including the wood-associated NAC domain transcription factors (PtrWNDs), BpNAC012, OsNAC29/31, and PpNAC1, demonstrated that they are functional orthologs of *Arabidopsis* SND1 and NST1 and are involved in the regulation of SCW biosynthesis [28,36]. The *PtrWND* genes are expressed in developing poplar wood. Expression of *PtrWNDs* in the *Arabidopsis snd1nst1* double mutant rescues the SCW defect [28]. In maritime pine, PpNAC1 acted as a main regulator of phenylalanine biosynthesis, and OsNAC29 and OsNAC31 regulate cellulose synthases genes (CESAs) in rice [37,38]. These studies indicated that VNSs from evolutionary distinct plant species participated in the regulation of SCW biosynthesis and that the VNS-mediated activation of the SCW biosynthetic program is highly conserved.

*Fragaria vesca*, the woodland strawberry, is emerging as a model plant for the cultivated octoploid strawberry as well as the Rosaceae family due to its small and sequenced genome, diploidy (chromosome number: 2n = 14; genome size = 240 Mbp), small stature, ease of growth, short life cycle, and ease of transformation [39,40,41,42,43]. The strawberry fruit is unique in that the edible flesh is actually an enlarged receptacle tissue. The true fruit are the numerous dry achenes spreading out of the receptacle’s surface. It offers an opportunity to investigate signal coordination and communication between different organs due to strawberry’s unique fruit structure [44]. The secondary wall thickening plays a great role in plant growth and development, but information about NAC transcription factors related to SCW thickening in fleshy fruits has not yet been investigated in detail. Therefore, studies on molecular mechanisms of SCW formation in strawberry will help us to understand the SCW thickening pathways and development cycling in fleshy fruits. Recently, FcNAC1 and FaNAC035/FaRIF were cloned in strawberry, respectively, which are involved in cell wall biosynthesis and fruit ripening [45]. FcNAC1 interacts with FcPL (*Fragaria chiloensis* pectin lyase) and contributes to cell wall remodeling [46]. Although FcNAC1 was reported to be clustered with VND family members in the phylogenetic analysis, whether it has the ability to induce the SCW thickening as members from other species has not been tested yet. FaRIF regulates ABA biosynthesis/signaling and cell wall degradation/modification [45]. Lignin synthesis is an important pathway regulated by VNSs. VNSs may have additional significance in strawberry fruit development besides regulators of SCW development, since biosynthesis pathway of lignin and anthocyanin, an important factor of color formation in strawberry fruit, share common precursor molecules [47,48]. Balance between lignin synthesis and anthocyanin synthesis needs to be well-coordinated for proper strawberry fruit development, and regulation towards VNSs may have roles in this process.

In this study, we isolated and characterized a VNS subfamily gene in *Fragaria vesca* and named *FvNST1b*. The sequence information, subcellular localization, and expression pattern of *FvNST1b* were investigated. Transgenic *Arabidopsis* plants overexpressing *FvNST1b* showed abnormal SCW thickening and induction of SCW-associated genes. Ectopic xylem cells were also produced by transient overexpression of *FvNST1b* in strawberry fruits. Our work demonstrated that FvNST1b of the NAC transcription factor family in strawberry possess conserved activity to promote SCW development, and may play critical roles in SCW formation in fruit.

## 2. Results

### 2.1. Cloning and Sequence Analysis of FvNST1b

**Figure 1:**
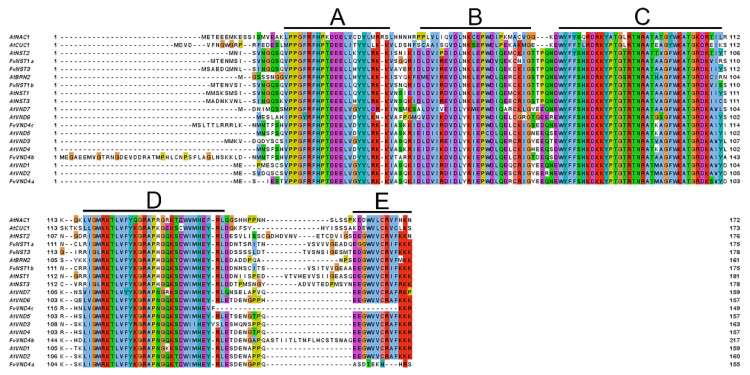
Amino acid sequences alignment of FvVNS and NAC proteins from *Arabidopsis* including AtVNSs (**A**–**E**). The proteins were initially aligned using Clustal omega. The NAC domain was marked with solid lines.

**Figure 2:**
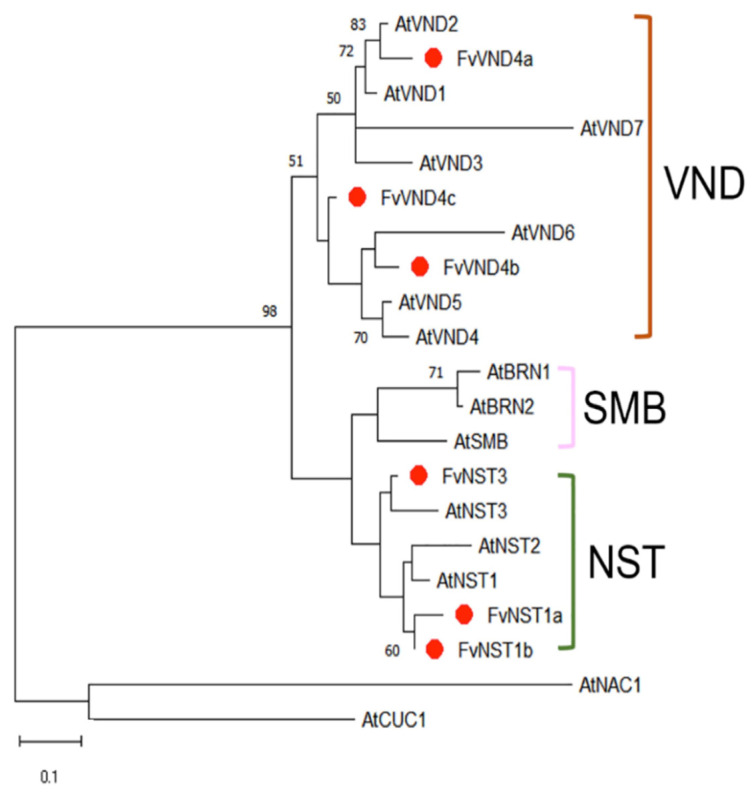
Phylogenetic tree of FvVNS and AtVNS proteins. The proteins were initially aligned using Clustal omega and then submitted for phylogenetic analysis using MEGA X software. The phylogenetic tree was constructed using the neighbor-joining method with 1000 bootstrap replications. Numbers indicate bootstrap values for the clades that received support values of over 50%.

Characterization of VNS genes from *Fragaria vesca* has not been performed yet. We identified *Fragaria vesca* VNS candidate genes from the SGR: Strawberry Genomic Resources database (http://bioinformatics.towson.edu/strawberry/; accessed on 1st November 2018). We performed multiple sequence alignment of *Arabidopsis* VNS family members and *Fragaria vesca* VNDs and NSTs. These protein sequences contain a highly conserved region towards the N-terminal, corresponding to the NAC domain, which is divided into five sub-domains, A to E (Figure 1). To investigate the relationship between FvVNSs and AtVNSs, a phylogenetic tree was constructed using their amino acid sequences. The phylogenetic tree indicated that all members are divided into VND, NST, and SMB subclades, with FvNST1b together with AtNST1 protein grouped into one cluster (Figure 2). The *FvNST1b* is annotated to encode a protein of 365 amino acids with an estimated molecular mass of 40.8 kDa and an isoelectric point of 6.27. These results suggested that FvNST1b is the closest counterpart protein of AtNST1, and a plausible candidate for the regulator of secondary wall thickening.

### 2.2. Subcellular Localization of FvNST1b Protein

**Figure 3:**
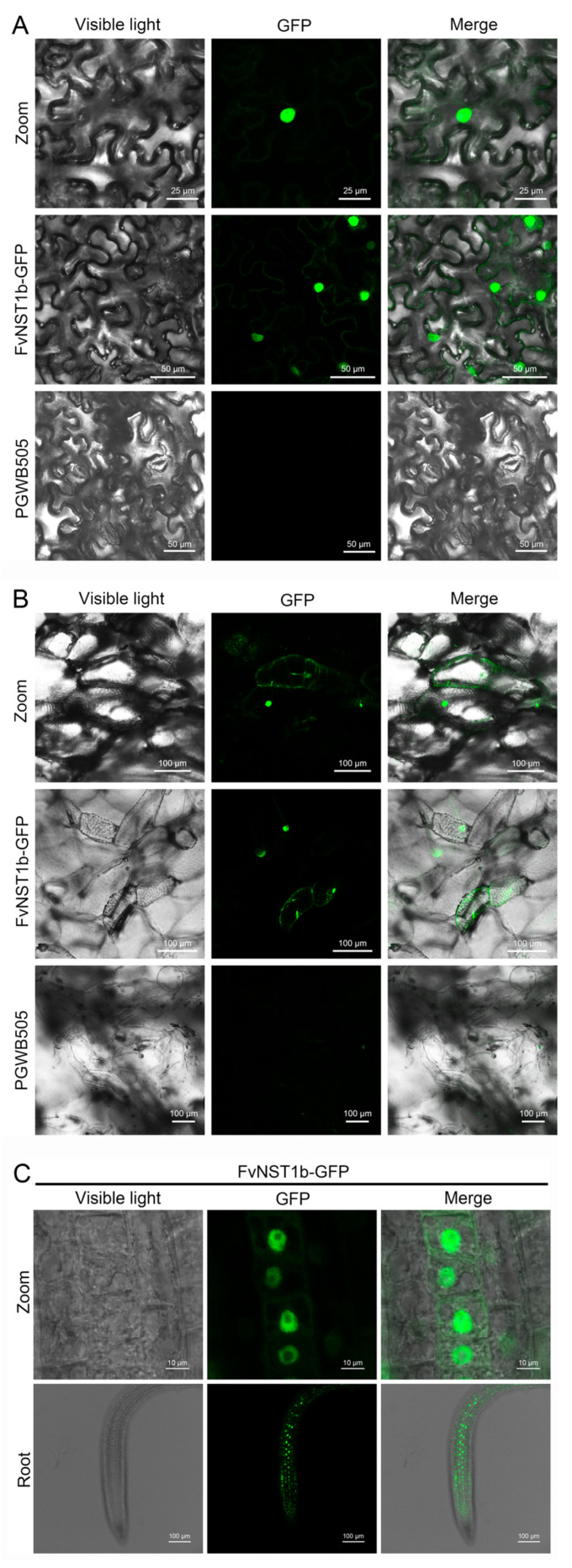
Subcellular localization of FvNST1b–GFP fusion protein. The tobacco leaf cells, strawberry fruit cells, and *Arabidopsis* were transformed with the plasmid FvNST1b–GFP or with the control vector with GFP. (**A**) Subcellular localization of FvNST1b-GFP fusion protein transiently expressed in tobacco leaf cells. The images of bright field, GFP and merged image of BF and GFP were shown. Scale bars represent 50 μm. (**B**) Subcellular localization of FvNST1b-GFP fusion protein in strawberry fruit cells. The images of bright field, GFP and merged were shown. Scale bars represent 100 μm. (**C**) Subcellular localization of FvNST1b-GFP fusion protein in stably transformed *Arabidopsis* root cells. The images of bright field, GFP and merged were shown. Scale bars represent 10 μm for zoom and 100 μm for root, respectively.

As a transcription factor, FvNST1b is expected to function in the nucleus. In order to examine its subcellular localization in vivo, we generated a vector containing coding region of *FvNST1b* fused with GFP reporter gene. The fusion gene plasmid and GFP control plasmid were transiently transformed into *Nicotiana Benthamiana* (hereafter tobacco) leaves and strawberry fruits. At 3 days after injection, a strong fluorescence signal was detected in the nucleus of tobacco leaf epidermal cells (Figure 3A), and strong GFP signal in nucleus was detected in the strawberry fruits at 4 days after injection (Figure 3B). In some cells, the GFP signal was also weakly detected in the surrounding area of the nucleus presumably in cytosol or ER that may represent FvNST1-GFP protein unsorted to the nucleus. At 7 days after induction, tobacco cells with ectopic striated cell walls are formed as in the overexpression of *AtNST1* reported by others (Supplemental Figure S1) [2]. Transient overexpression of FvNST3-GFP also induced similar effects (Supplemental Figure S1). We also generated stably transformed *Arabidopsis* plants with *FvNST1-GFP*. *Arabidopsis* transgenic seedlings also showed strong nuclear-localized GFP signals and weak cytoplasmic/ER signals in roots (Figure 3C).

### 2.3. Expression Analysis of the FvNST1b Gene

**Figure 4:**
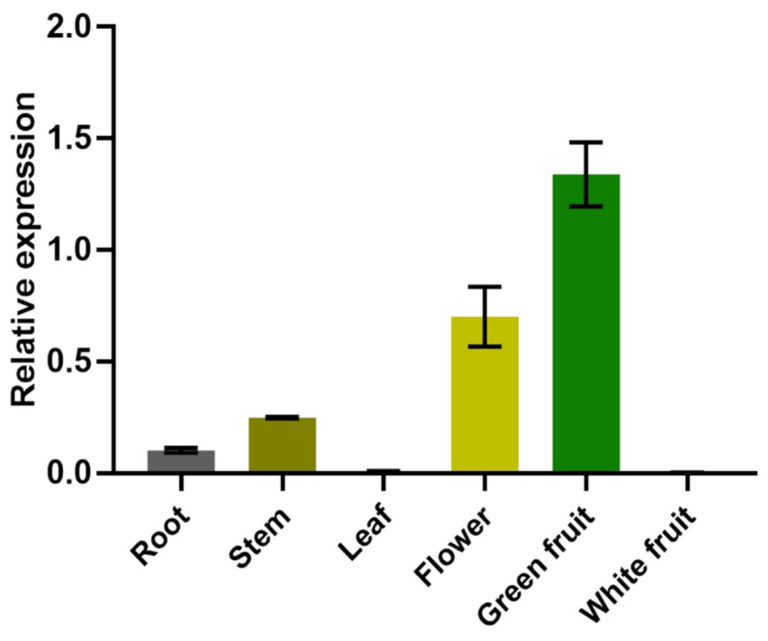
Analysis on tissue expression pattern of FvNST1b. Each column represented the mean of three replicates. Error bars on each column represented the standard error (S.E.) of three replicates.

Tissue-specific expression analysis of *FvNST1b* was performed by qRT-PCR using various tissues from strawberry plants. *FvNST1b* displayed a differential expression pattern in *F. vesca* (Figure 4). *FvNST1b* transcripts were almost undetectable in leaf and white fruit, and its expression was detected in vegetative parts of strawberry at relatively low levels, including in roots and stems, whereas its expression in flowers and green fruits are significantly higher, with the highest level in green fruits. This observation suggested that *FvNST1b* mainly function in the fruits of strawberry at the earlier developmental stages.

### 2.4. Overexpression of FvNST1b Induces Ectopic Thickening of Secondary Walls in Various Tissues of A. thaliana

**Figure 5:**
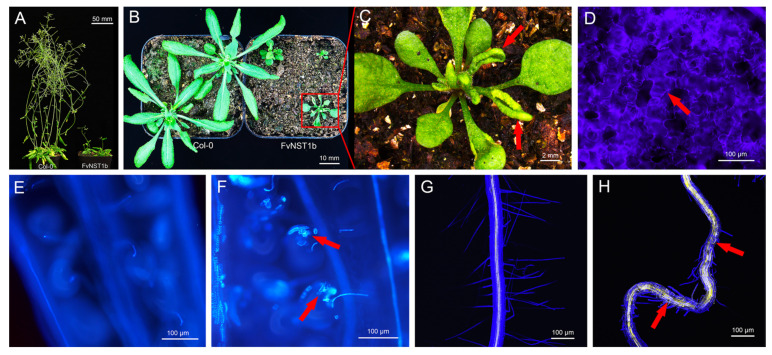
Overexpression of *FvNST1b-GFP* in *Arabidopsis* caused abnormal plant development and ectopic deposition of SCW. (**A**) WT and *35S:FvNST1b-GFP* plants grow on soil for 40 days. *35S:FvNST1b-GFP* plants grew more slowly than wild-type plants. (**B**) WT and *35S:FvNST1b-GFP* plants grow on soil for 21 days. *35S:FvNST1b-GFP* plants showing upwardly curled rosette leaves. (**C**) Magnified view of *35S:FvNST1b-GFP* plant in (**B**). (**D**) Leaf epidermis of a *35S:FvNST1b-GFP* plant under UV illumination. (**E**) Ovules of Wild-type plant under UV illumination. (**F**) Ovules of a *35S:FvNST1b-GFP* plant under UV illumination. (**G**) Wild-type root stained with Calcofluor White and Auramine O. (**H**) A root from a *35S:FvNST1b-GFP* plant stained with Calcofluor White and Auramine O. Root was abnormally bent and ectopic lignified secondary wall thickening is apparent as Auramine O fluorescence signal (yellow signal, red allows). Scale bars represent 50 mm for (**A**), 10 mm for (**B**), 2 mm for (**C**), and 100 μm for (**D**–**H**).

In order to test if *FvNST1b* has the ability to promote SCW deposition, we expressed *FvNST1b-GFP* ectopically under the control of the CaMV35S promoter (*35S:FvNST1b-GFP*) in transgenic *Arabidopsis* plants. The *35S:FvNST1-GFP b* plants were usually smaller and grew more slowly than wild-type plants. Ectopic expression of *FvNST1b-GFP* induced ectopic lignified secondary wall thickening in various tissues, including anthers, stamens, ovules, stems, leaves, and root tissues as reported for the overexpression of *Arabidopsis* NST1 [2]. Epidermal cells with ectopic secondary wall thickening typically had a striated appearance similar to that of tracheary elements (Figure 5).

These observations suggest that the abnormal appearance of leaves and floral organs of *35S:FvNST1b-GFP* plants was due to the ectopic accumulation of lignified materials, reflecting previous reports that NSTs are regulators of secondary wall thickening in various tissues.

### 2.5. Overexpression of FvNST1b Induces SCW Formation in Strawberry Fruits

**Figure 6:**
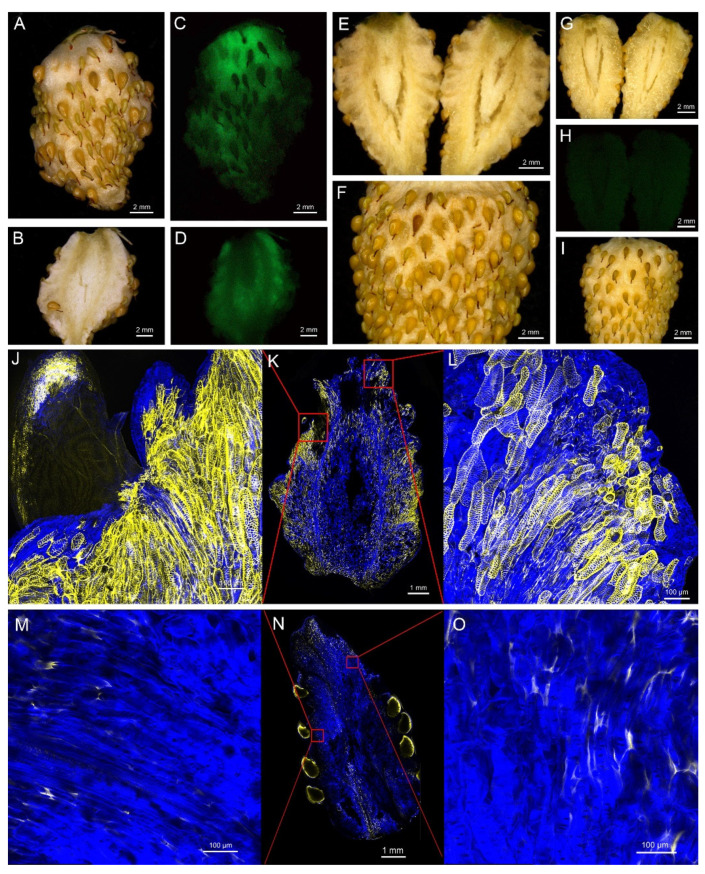
Overexpression of *FvNST1b* in strawberry fruits induced ectopic deposition of SCW. (**A**,**B**) A whole fruit (**A**) and a section image (**B**) of strawberry fruit at four days after Agrobacterium infection of *35S:FvNST1b-GFP*. (**C**,**D**) GFP image of a whole strawberry fruit (**C**) and a section (**D**) of a strawberry fruit at four days after Agrobacterium infection of *35S:FvNST1b-GFP*. GFP signal is detected in most part of fruit sample. (**E**) Section image of strawberry fruit at five days after Agrobacterium infection of *35S:FvNST1b-GFP*. (**F**) Close-up view of strawberry fruit at five days after Agrobacterium infection of *35S:FvNST1b-GFP*. (**G**) Section image of strawberry fruit at five days after Agrobacterium infection of empty vector. (**H**) GFP image of strawberry fruit at five days after Agrobacterium infection of empty vector without detectable GFP signal. (**I**) Fruit image at five days after Agrobacterium infection of empty vector. (**J**–**L**) Images of hand-sectioned *35S:FvNST1b* infected fruit stained with Calcofluor White and Auramine O. Ectopic lignified secondary wall thickening is apparent as yellow signal in fruit cells. (**J**,**L**) is a magnified image of (**K**), with positions indicated as squares. (**M**–**O**) Images of hand-sectioned empty vector infected fruit stained with Calcofluor White and Auramine O. (**M**,**O**) is a magnified image of (**N**) with positions indicated as squares. Scale bars represent 2 mm for (**A**–**I**), 100 μm for (**J**,**L**,**M**,**O**), and 1 mm for (**K**,**N**).

To further confirm that *FvNST1b* has the ability to promote SCW deposition in strawberry fruit, we transiently overexpressed the *FvNST1b* gene by using the agrobacterium infiltration into S7 fruits of *Fragaria vesca* (Figure 6), which is at the green stage and make the transition to ripening [49]. After four days from infiltration, the *35S:FvNST1b-GFP* infiltrated strawberry fruits exhibited GFP signals (Figure 6A–D). After five days, the *35S:FvNST1b-GFP* infiltrated strawberry fruits exhibited enhanced lignification phenotypes, along with many cells having striated appearance similar to that of tracheary elements (Figure 6E,F,J–L). The *35S:FvNST1b-GFP* infiltrated fruits tended to be more pliable and the space among seeds closer than that of 5d after the injection of PGWB505 vector-control infiltrated fruits (Figure 6G–I,M–O). Longitudinal sections of strawberry fruits stained for lignin and cell wall structure confirmed that overexpression of *FvNST1b-GFP* resulted in excessive SCW deposition in strawberry fruit cells, indicating their ability to promote SCW formation in strawberry fruits.

### 2.6. Enhanced Gene Expression of SCW Related Genes in 35S:FvNST1b Transgenic Arabidopsis Plants

**Figure 7:**
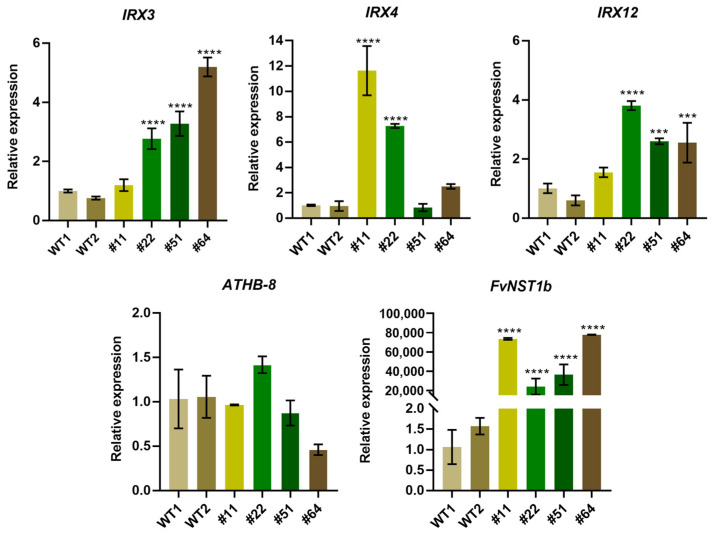
Induction of expression of genes related to the differentiation of tracheary elements in transgenic *Arabidopsis* plants overexpressing *FvNST1b-GFP*. Expression of genes related to the differentiation of tracheary elements were analyzed in 2 WT plants as controls and 4 independent transgenic *Arabidopsis* lines overexpressing *FvNST1b-GFP* by quantitative RT-PCR. Each bar represents the amount of the transcript of a gene relative to that of the internal control. Error bars represent ± SD (n = 3). Asterisk indicate a significant difference compared to the WT1 by *t*-test (****, *p* < 0.0001; ***, *p* < 0.001).

To further prove the ability of *FvNST1b* to promote SCW development as in the orthologous genes from other species such as AtNST1, we examined the effect of *FvNST1b-GFP* overexpression in *Arabidopsis* on gene expression of known downstream genes for AtNST1 (Figure 7). We examined the expression of *IRREGULAR XYLEM3 (IRX3;* encodes a cellulose synthase*)*, *IRX4* (encodes a cinnamoyl CoA reductase), *IRX12* (encodes a putative laccase) as genes known to be upregulated by AtNST1 overexpression [50,51,52], and *HOMEOBOX GENE8 (ATHB-8)*, which is involved in the vascular developmental process upstream of VNDs/NSTs [53].

The expression of the *IRX3*, *IRX4*, and *IRX12* genes was enhanced 1- to 10-fold in all four of the independent *35S:FvNST1b-GFP* transgenic lines examined, as compared with the wild type. In contrast, *ATHB-8* was not upregulated in *35S:FvNST1b-GFP* plants. Our results suggest that *FvNST1b* has the ability to function as a crucial regulator for secondary wall thickening by inducing key downstream genes similar to their counterpart transcription factors in other plant species.

## 3. Discussion

Strawberry is one of the most economically important fruit crops and has been considered a genuine example of a plant showing non-climacteric fruit ripening [54,55]. The ripe strawberry fruits undergo continual softening and easily become rotten. Thus, improvement of strawberry shelf life has become an important factor in current breeding programs, even when these quality attributes are controlled by a complex genetic background [56]. Secondary wall thickening provides mechanical support for various plant tissues, and thus the SCW formation may contribute to fruit firmness [25,57]. NSTs/VNDs are master transcriptional switches regulating the developmental program of SCW biosynthesis by activating downstream transcription factors [25,58]. Although NAC transcription factors and the lignin biosynthesis have been studied in strawberry fruit development recently [45,46], their contribution regarding the molecular control of secondary wall thickening is largely unknown. In the present study, we described the isolation and characterization of *FvNST1b*, an NST1-like homolog from *Fragaria vesca*.

Amino acid sequence alignment and phylogenetic analysis suggested that *FvNST1b* is a member of the NST class of NACs. Plant NAC domain proteins are one of the largest groups of plant-specific transcriptional factors and have been reported to participate in many developmental processes, including SCW formation and biotic and abiotic stress responses [59,60]. Amino acid sequences of NAC proteins typically contain an NAC domain; five highly conserved subdomains at the N-terminal. The five highly conserved subdomains are also present in the FvNST1b sequence. Our phylogenetic analysis placed FvNST1a and FvNST1b as the closest homolog of *Arabidopsis* NST1 and NST2. Indeed, results of our overexpression experiments suggested that *FvNST1b* and *FvNST3* have the ability to promote SCW development as in *Arabidopsis* NSTs. In our phylogenetic analysis, we could not find counterparts for some members of the VND-related *Arabidopsis* proteins, namely SOMBRERO (SMB), BEARSKIN1 (BRN1), and BRN2 among strawberry VNS candidates [35]. Those proteins still have the ability to induce ectopic SCW deposition when overexpressed, but they are involved in root cap development in *Arabidopsis* [34,35]. It is speculated that there may be other unidentified members of the VND family or other transcription factors in strawberry to regulate the development of strawberry root caps.

*FvNST1b* is expressed preferentially in strawberry green fruit, suggesting that it has important roles in the regulation of strawberry fruit development. Overexpression of *FvNST1b* in *Arabidopsis* caused ectopic deposition of SCW (Figure 5), in agreement with previous studies. In addition, overexpression of *FvNST1b* in strawberry fruits also caused ectopic deposition of SCW along with lignin accumulation, fruit shrinkage, and fruit color change. Anthocyanin contributes to the fruit color in strawberry. Biosynthetic pathways for lignin and flavonoids, including anthocyanin, share common precursors from the general phenylpropanoid pathway [48]. Several TF genes in the MYB family are reported to co-repress or co-activate genes involved in the biosynthesis of lignin and flavonoids. There are also cases of regulation towards lignin or flavonoid synthesis to achieve proper balance of carbon flow between lignin and flavonoids. Some of those members such as AtMYB20 are reported to be under regulation by VNSs [61]. Thus, VNSs in strawberry including FvNST1b may contribute to co-regulate and/or balance lignin deposition and anthocyanin synthesis, which is a crucial factor for strawberry fruit quality and commercial value. Future analysis of contribution of VNSs on the regulation of flavonoid-synthesis-related genes will be important to further prove this idea.

A nuclear localization signal was predicted within *FvNST1b* amino acid sequences, and the transient transformation of tobacco and strawberry fruit cells with *FvNST1b* fused to a reporter gene showed that FvNST1-GFP localizes to the nucleus. Furthermore, nuclear localization was detected in *35S:FvNST1b-GFP* transgenic *Arabidopsis* plants. Other members of this NAC family also exhibited nuclear localization: an NAC from *S. lycopersicum* was shown to be located in the nucleus when ectopically expressed in onion epidermal cells, as was also the case for AtNAC2 [62]. MtNST1 from alfalfa, described as a SCW master switch, was also identified in the nucleus of epidermal tobacco cells [63]. The tomato SlNAC3 has been localized in the nucleus of onion epidermal cells by transient expression analysis [62]. Seven GhSWN proteins from cotton all located in the nucleus and were consistent with their functions as transcription factors [27]. In order to test whether *FvNST1b* has ability to activate SCW-related genes as in AtNSTs, the expression of *IRX3*, *IRX4*, *IRX12,* and *ATHB-8* were examined in *35S:FvNST1b* transgenic plants, which are involved in the differentiation of tracheary elements upstream or downstream of AtNSTs [2]. *IRX3*, *IRX4,* and *IRX12* were upregulated in *35S:FvNST1b* transgenic plants. In contrast, *ATHB-8* was not regulated in *35S:FvNST1b* plants, which is in line with previous reports in *Arabidopsis*. These results show that *FvNST1b* is a positive regulator of secondary wall thickening. Induction of downstream genes of AtNST1 in transgenic *35S:FvNST1b Arabidopsis* plants further support the idea that FvNST1b acts as a transcriptional factor to regulate downstream processes of SCW development.

In summary, an NST1-like gene, *FvNST1b*, was isolated and characterized from strawberry. *FvNST1b* has high sequence similarity to other NSTs homologs and contained the well-conserved NAC domain. The FvNST1b protein mainly localizes in the nucleus. *FvNST1b* is highly expressed in young fruit. In addition, overexpression of *FvNST1b* caused ectopic deposition of SCW and upregulated the expression of genes related to the differentiation of tracheary elements such as *IRX3*, *IRX4,* and *IRX12* in transgenic *Arabidopsis*. Moreover, overexpression of *FvNST1b* in strawberry fruits also caused ectopic deposition of SCW along with lignin accumulation and fruit shrinkage. These results suggest that FvNST1b is a transcription factor promoting SCW thickening in strawberry. Although we were unable to detect any expression of *FvNST1a* in strawberry fruit, we also showed that at least another closely related member, *FvNST3,* which is expressed in fruit, has a similar function. Functional redundancy as well as their specialization will be explored in the future. The evidence provided will contribute to understanding the regulatory network that takes place during the development and ripening of strawberry fruit.

## 4. Materials and Methods

### 4.1. Plant Material and Growth Conditions

Diploid strawberry plants (*Fragaria vesca*), Yellow Wonder 5AF7 (YW5AF7) [40], planted in pots (90 mm × 90 mm × 90 mm) were used in this study. The seedlings were grown and maintained in a growth room with the following conditions: 22 °C, 60% humidity, and a 16-h photoperiod. Hand pollination was performed by using downy water bird feather to obtain pollinated fruit. Samples of root, stem, leaf, flower, and fruit were collected for tissue-specific expression assays. For *Arabidopsis* transformation, *Arabidopsis thaliana* (ecotype Columbia) was used and grown in soil at 22 °C with 16 h of light daily.

### 4.2. DNA Preparation and Gene Cloning

Genomic DNA (gDNA) of strawberry samples was isolated by the CTAB method. To clone the *FvNST1b* gene, the AtNST1 protein was used for a BLAST search in the strawberry genome GBrowse (http://www.strawberrygenome.org/ (accessed on 19 October 2022)), and a high homology protein with the gene locus101309102 was found. Then, the specific primers for full-length of DNA cloning were designed for *FvNST1b* (forward, 5′-attB1-ATG ACT GAA AAC GTG AGC AT-3′; reverse, 5′-attB2-TTA TAT ATG ACC ATT CGA CAC GTG-3′) and *FvNST3* (forward, 5′-attB1-ATG TCT GCA GAG GAT CAA ATG-3′; reverse, 5′-attB2- TTA TAC CGA CAG GTG GCA TAA TG-3′) using the SnapGene program (https://www.snapgene.com/ (accessed on 19 October 2022)). PCR was performed using Primer Start Max Enzyme (TaKaRa Biotech, Dalian, China) under the following conditions: 98 °C for 30 s, followed by 34 cycles at 98 °C for 10 s, 55 °C for 15 s, and 72 °C for 30 s.

### 4.3. Bioinformatics Analysis

The amino acid sequence of FvNST1b was downloaded in JGI (https://phytozome-next.jgi.doe.gov/ (accessed on 19 October 2022)). Protein sequences were aligned using clustal omega (https://www.ebi.ac.uk/Tools/msa/clustalo/ (accessed on 19 October 2022)) and the alignment was edited with Jalview (https://www.jalview.org/ (accessed on 19 October 2022)). The phylogenetic tree was constructed with the neighbor-joining method using MEGA X software. Bootstrap support percentages were calculated from 1000 replications.

### 4.4. Construction of Plasmid DNA

The GATEWAY™ conversion technology (Invitrogen, Gaithersburg, MD, USA) was used in the experiment. To generate the *FvNST1b* overexpression vector, full-length *FvNST1b* DNA (1541 bp) was amplified and inserted into the PDONR221 vector under the treatment of BP Enzyme (Invitrogen). The entry vector DNAs were transformed into *Escherichia coli* DH5α cells and sequenced. The PDONR221-*FvNST1b* was treated with LR Enzyme (Invitrogen) and cloned into the PGWB505 vector containing the GFP reporter gene to generate *PGWB505-FvNST1b-GFP.*

### 4.5. Transient Expression of FvNST1b in Nicotiana Benthamiana Leaves and Sub-Cellular Localization Analysis

*PGWB505-FvNST1b* vector was introduced into *Agrobacterium tumefaciens* strain GV3101 by thermal shock in liquid nitrogen. Transformed bacteria were plated on a selective medium yeast mold agar containing kanamycin, hygromycin, and rifampicin at a final concentration of 100 µg/mL each. Resistant colonies were analyzed by PCR for the presence of full-length *FvNST1b* gene using the primers mentioned above. A positive colony was cultured in selective LB liquid medium and incubated at 28 °C until an O.D.600 between 0.8 and 1.0, and then the cells were resuspended in infection buffer and shaken for 2 h at 28 °C. A 1-mL syringe was used to inject the agrobacterium suspension into the abaxial face of young tobacco leaves (two weeks old), and samples were analyzed after two days of infiltration. Subcellular localization of *FvNST1b* in transient-transformed leaf samples was analyzed through visualization of the tissue under a confocal fluorescence microscope (Leica Confocal microscope SP8X; Leica Microsystems GmbH, Wetzlar, Germany ) with a 10*×* objective lens, a 488 nm laser from tunable white light laser for excitation, and a 499 nm to 551 nm bandwidth for detection.

### 4.6. Gene Expression Level Analysis

Total RNA from the strawberry samples was extracted using the polysaccharide and polyphenolics-rich RNAprep Pure Kit (Tiangen, Beijing, China); cDNA was synthesized from total RNA using the PrimeScript RT reagent Kit (Perfect Real Time) (Takara). Total RNA of *Arabidopsis* was extracted using the PLANT RNA Kit (omega). The cDNA samples were diluted 1:5 with water; 2 μL of the diluted cDNA was used as a template for quantitative real-time PCR (qRT-PCR) analysis. Real-time quantitative PCR was performed in the ABI 7500 Real-Time PCR System (Applied Biosystems, Waltham, MA, USA) using SYBR Premix Ex Taq II (Takara). The PCR program included an initial denaturation step at 95 °C for 3 min, followed by 40 cycles of 10 s at 95 °C, and 30 s at 57 °C. Each sample represented three biological replicates; each of them included four technical replicates. The relative expression levels of target genes were calculated with the formula 2^−ΔΔCT^ in strawberry and 2^−ΔCT^ in *Arabidopsis*.

In strawberry, the *Actin* gene was used as the internal control. The gene-specific primers used were as follows: 5′-GCC AGA AAG ATG CTT ATG TCG GTG-3′ (forward) and 5′-TGG GGC AAC ACG AAG CTC AT-3′ (reverse) for *Actin* as in the previous publication from others [49]; 5′- ATG GTT GTT GGA TTG GAC TTT GG-3′ (forward) and 5′-TAG ACG TAA CAC CCT GCC TGC-3′ (reverse) for *FvNST1b* with primer efficiency of 95.3% calculated by 1:10 serial dilution standard curve containing 5 points. In *Arabidopsis*, the internal control Actin gene was used in the qRT-PCR analysis. The gene-specific primers used were as follows according to a previous publication from others [2]: 5′- CCA CAT GCT ATT CTG CGT TTG GAC C -3′ (forward) and 5′- CAT CCC TTA CGA TTT CAC GCT CTG C-3′ (reverse) for *Actin*; 5′-GGC AAA CTC AAG TGG CTT GAG CG-3′ (forward) and 5′-TAA CTC CGC TCC ATC TCA ATT CC-3′ (reverse) for *IRX3*; 5′-CGT TAT CTC CTA GCC GAG AGT GCT C-3′ (forward) and 5′-TGC CAT TTT CCA CGG ATT CTT GCG ATG C-3′ (reverse) for *IRX4*; 5′-GGT GGA TGG GTC GTC ATG AGA TTC-3′ (forward) and 5′-CGT GGC GTG ATG TTG ATA TGT CGC CC-3′ (reverse) for *IRX12*; 5′-TGT GTT GCT CAC TCA AGG CCT TA-3′ (forward) and 5′-TGT CGA AGA TCT TGT CGA GAG TGA-3′ (reverse) for *AtHB-8*.

### 4.7. Arabidopsis Transformation

*Agrobacterium tumefaciens* strain mentioned earlier were transformed into wild-type *Arabidopsis* plants using the floral dip method. Transgenic seedlings were selected on half-strength Murashige and Skoog (MS) agar plates containing 50 mg/L hygromycin and 200 mg/L Timentin; antibiotic-resistant plants were then tested by GFP signal and separation ratio to confirm the presence of the transgene. Four independent lines of the T3 generation were randomly chosen for further analysis.

### 4.8. Transient Overexpression of FvNST1b in Strawberry Fruit

*Agrobacterium tumefaciens* strain GV3101 mentioned above was used to perform transient expression analyses in strawberry fruits [64]. For *Agrobacterium* infection, the Agrobacterium suspension was injected into the fruit using a syringe of 1 mL capacity. To do this, the needle tip was inserted into the fruit center from the top, and then the Agrobacterium suspension was slowly and evenly injected into the fruits until the strawberry fruit was completely infected. After the infection, the fruits were incubated under the conditions required for the different experimental aims. The effect of overexpression was evaluated by examining the changes in both reporter gene expression and related phenotypes after Agrobacterium infection.

### 4.9. Fruit Sections and Staining

The infected strawberry fruits were embedded in 10% agarose gel at 7 days after, and 200 µm thick sections were cut with a vibratome. Strawberry fruit sections and *Arabidopsis* seedlings were fixed with 4% PFA for 60–120 min at 23–25 °C temperature with vacuum treatment. After fixation, the materials were washed twice for 1 min in 1 × PBS and moved to the clearing solution. After rinsing in 1 × PBS, the plant material was transferred to the ClearSee solution [65] and cleared overnight at room temperature. We prepared 0.1% Auramine O in ClearSee solution and the materials were stained overnight. Then, the materials were washed for at least 1 h with gentle shaking. The materials were transferred to 0.1% Calcofluor White in ClearSee solution and stained for 30 min; the materials were washed in ClearSee for 30 min with gentle shaking. Materials were analyzed with Leica TCS SP8X inverted confocal microscope. Imaging Calcofluor White was performed by a 405-nm diode laser for excitation and detection band width at 425–475 nm. Imaging Auramine O was performed with 488 nm from a tunable white light laser and band width detected at 505–530 nm.

## Supplementary Materials

The following supporting information can be downloaded at: https://www.mdpi.com/article/10.3390/ijms232113212/s1.
