# Supplementary material for: FvNST1b NAC Protein Induces Secondary Cell Wall Formation in Strawberry
Source: Int J Mol Sci. 2022 Oct 30;23(21):13212. doi: 10.3390/ijms232113212 (PMC9654860; doi:10.3390/ijms232113212)
Supplement: Supplementary file 1 [file ijms-23-13212-s001.zip › ijms-1882983-supplementary.pdf]

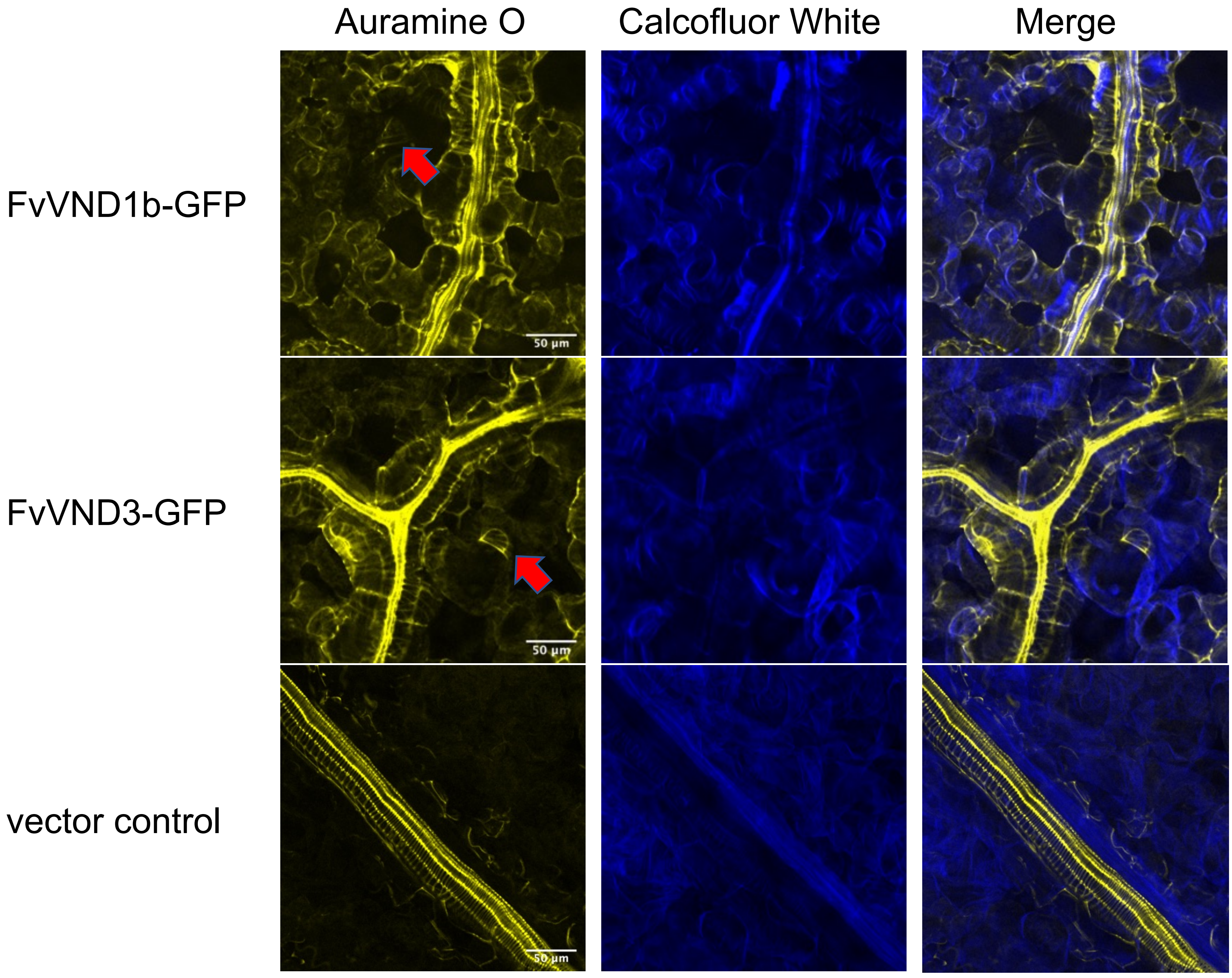

Supplementary Figure S1. Transient overexpression of FvNST1b or FvNST3 induce enhanced lignification in tobacco leaf

Images of tobacco leaves 7days after Agrobacterium infection of either *35S:FvNST1b-GFP, 35S:FvNST3-GFP,* or empty vector. Leaves are stained with Calcofluor White and Auramine O. Ectopic lignified secondary wall thickening is apparent in leaves infected with *35S:FvNST1b-GFP, 35S:FvNST3-GFP* (red arrows). Scale bars represent 50 µm
